# Standardization of molecular techniques for the detection and characterization of intestinal protozoa and other pathogens in humans

**DOI:** 10.1590/1678-9199-JVATITD-2021-0099

**Published:** 2022-05-06

**Authors:** Maria Alejandra Vethencourt Ysea, Mariana Cedeño Umaña, Sofia Pereira Fuentes, Idalia Valerio Campos, Misael Chinchilla Carmona

**Affiliations:** 1 University of Medical Sciences, Laboratory of Basic Research, San José, Costa Rica.; 2 University of Medical Sciences, Faculty of of Microbiology, San José, Costa Rica.

**Keywords:** Molecular detection, Molecular characterization, PCR-RFLP, Intestinal protozoa, *Blastocystis* spp., Costa Rica

## Abstract

**Background:**

The intrinsic sensitivity limitations of basic parasitological methods, along with the particular biological characteristics of parasites, make these methods ineffective to differentiate morphologically indistinguishable species. Molecular detection and characterization techniques could be used to overcome these problems. The purpose of this work was to standardize molecular polymerase chain reaction (PCR) techniques, described in the literature, for the detection and molecular characterization of intestinal protozoa and other pathogens in humans.

**Methods:**

DNA was extracted from human or animal feces, previously washed or cultured in Boeck Drbohlav's Modified Medium. DNA extraction was performed with Machery-Nagel extraction kits. The standardization of the PCR, nested-PCR or RFLP techniques was carried out according to the literature. For each molecular technique performed, the sensitivity of the test was determined based on the minimun quantity required of DNA (sensitivity A) and the minimum quantity of life forms that the test detected (sensitivity B).

**Results:**

Sensitivity A was 10 fg for *G. duodenalis*, 12.5 pg for *Entamoeba histolytica* or *Entamoeba dispar*, 50 fg for *Cryptosporidium* spp., 225 pg for *Cyclospora* spp. and 800 fg or 8 fg for *Blastocystis* spp. after performing a 1780 bp PCR or 310 bp nested PCR, respectively. The sensitivity B was 100 cysts for *G. duodenalis,* 500 cysts for *E. histolytica* or *E. dispar*, 1000 oocysts for *Cyclospora* spp. and 3600 or four vegetatives forms for PCR or nested PCR of *Blastocystis* spp., respectively.

**Conclusions:**

The molecular detection of protozoa and chromist was achieved and the molecular characterization allowed the genotyping of some of the parasites such as *Giardia duodenalis*, *Cryptosporidium* spp., and *Blastocystis* spp. This study summarizes the molecular techniques for epidemiological studies in humans and animals, and helps in the investigation of their transmission sources in countries where intestinal parasites are a public health problem.

## Background

Intestinal human parasites have a worldwide distribution. They can be protozoa and helminths and are a public health problem that affects people of all ages, especially children and the elderly, particularly in least developed countries [1]. According to the 2008-2009 National Health Survey [2], the prevalence of *Blastocystis* spp. in Costa Rica was around 37.8%, increasing with age to 46.6%. *Entamoeba histolytica*/ *E. dispar* leads the list among the pathogenic protozoans with 3.2% followed by *Giardia duodenalis* with 2.9% already. The eradication of intestinal helminths in Costa Rica was recorded [3]; however, in more recent studies carried out by Abrahams-Sandi *et al*. [4] and Arévalo *et al*. [5], higher prevalences of helminths and of pathogenic protozoa were present in children in Limón [4] and Goicochea [5]. According to the 2008-2009 National Survey [2], the prevalence of parasites in Costa Rica is closely related to poor socio-economic and hygienic characteristics [6].

Parasitic bowel diseases cause low mortality; however, the easy transmission way, the chronicity of symptoms, and the associated potential complications are important public health and sanitary problems. Some intestinal parasites can go unnoticed without producing symptoms, but they can also cause digestive symptoms of varying intensity, even with serious repercussions on the body [7]. They can affect the individual’s productivity or educational performance, causing absenteeism from work, and anthropometric nutritional status in infected school-age children [7].

Detection techniques that are based on basic parasitological methods such as direct examination, concentration or culture methods have sensitivity limitations. Moreover, the particular biological characteristics of the parasites make them more inefficient when it comes to detecting low counts or to differentiate species that are morphologically indistinguishable [8,9]. Cases like these require the sensitivity characteristics of molecular biology techniques.

The application of molecular techniques to detect and identify the DNA of the parasite sets the scene for the molecular epidemiology of intestinal parasitosis, especially for pathogens that are morphologically indistinguishable from commensal species. An example of this would be the differentiation of *Entamoeba histolytica* (pathogen) from the commensal species (*Entamoeba dispar* or *Entamoeba moshkovskii*) morphologically indistinguishable [10]. In the case of the genus *Blastocystis* spp., genotypes are morphologically indistinguishable, but some of them have been related to pathogenicity [11]. Molecular biology allows the detection *Cryptosporidium* spp., a small size protozoan that requires special staining, such as the modified Ziehl Neelsen [12] technique, to observe its presence. The use of molecular tools would increase the detection level to make it possible to identify the presence of these species. Currently, 42 species of *Cryptosporidium* are recognized [13], with *Cryptosporidium parvum* and *Cryptosporidium hominis* being responsible for more than 90% of human infections [14]. *C. hominis* has been linked to anthroponotic tranmission [15] whereas *C. parvum* presents a zoonotic transmission route with livestock as the primary source of infection [16]. Therefore, the application of molecular techniques would allow the identification of human pathogenic intestinal protozoa, increase the detection limit, and even improve the characterization of species or genotypes. 

In this work, a compendium of molecular biology techniques that allow the detection and molecular characterization of the genus and species or genotypes of protozoa pathogens in humans is presented. Moreover, the detection limit of evolutionary life forms of *E. histolytica* and/or *E. dispar* and/or *E. moshkovskii*, *G. duodenalis*, *Cryptosporidium* spp., *Cyclospora* spp. and stramenopiles such as *Blastocystis* spp. is also suggested as a molecular detection tool for parasites.

The present study paves the way for the molecular epidemiology of intestinal protozoosis and *Blastocystis* spp., which may serve for epidemiological studies in humans and animals, the investigation of the sources of transmission of some of these parasites and as diagnostic tools, which may be used at the service of the population.

## Methods

### Obtaining positive controls for molecular tests

The parasites to be detected molecularly were obtained from human and animal feces, donated to UCIMED Basic Research Laboratory (LIB-UCIMED). Direct parasitological analysis was performed on the samples with 0.85% saline and lugol [17] looking for protozoan cysts or with permanent stains such as modified Koster to detect *Cryptosporidium* spp. [18], or Ziehl Nielsen to observe the presence of *Cyclospora* spp. [19]. The feces samples with cysts, were washed (HL) to eliminate the excess of contaminants inherent in the sample, resuspending 1 g of the sample in 10 mL sterile warm distilled water, macerating with wooden sticks and centrifuged at 1750 x *g*/10 min (Frontier™ Serie 5000, OHAUS, USA). The procedure was carried out as many times as necessary to obtain a clear supernatant. The final pellet was resuspended in 5 mL of sterile distilled water, aliquoted at a rate of 1 mL in 1.5 mL ependorf tubes, centrifuged at 21380 x *g*/2 min (Mikro 200, Hettich Instruments) and stored at -20 °C until DNA extraction. DNA extraction was performed with the Machery-Nagel Extraction Kit (NucleoSpin® Tissue). Feces samples positive for *Blastocystis* spp. (vegetative forms and/or cysts) were cultivated in Modified Boeck Drbohlav's Medium (MBDM) for up to 96 hours to increase the vegetative forms of this parasite [17]. Finally, the pellet was washed 3 times with Ringer's buffer by centrifugation at 1750 × *g*/10 min, and subreadsequently stored at -20° C until use. 

### DNA extraction

The DNA extraction was carried out with the Machery-Nagel extraction kits (NucleoSpin® Tissue), standardizing the mechanical lysis process with and without the presence of glass beads, according to the suggestions of Sepahvand *et al*. [20]. Briefly, the pellet stored at -20° C was resuspended with 250 µL of TE buffer (10 mM Tris/HCl; 1 mM EDTA, pH 8) plus 200 mg of cover glass powder #1, sterile. The lysis process was carried out three times. Each cycle consisted of cooling the preparation for 3 min at 4° C in a thermal block (Torrey Pines Scientic, USA) and mixing for 3 min in vortex. Then, it was centrifuged for 1 min at 21380 x *g*/2 min (Mikro 200, Hettich Instruments), the supernatant was transferred to a clean tube, and the DNA extraction was continued with the Machery-Nagel Kits (NucleoSpin® Tissue), following the indications of the commercial company recommended for the extraction of eukaryotic cells. 

### Measurement of DNA quality

DNA was quantified with the Qubit fluorometer (ThermoFisher, USA), following the manufacturer's instructions. The integrity of the DNA (10 µL), with 2 µL of the 6X loading buffer (6X MassRuler, Loading Dye Solution, Fermentas) was evaluated after carrying out an agarose gel electrophoresis (SeaKem LE, Cambrex, USA) at 1%, dissolved in Tris-Acetate-EDTA Buffer (TAE), stained with GelRed (Gel Stain, Biotium, Cat: 41003). Electrophoresis was performed at 100 volts (FB1000 Power Source, Fisher Scientific, USA). To visualize the PCR amplifications and the RFLPs, electrophoresis was carried out on a 2% agarose gel, following the same methodology. The size of the amplified PCR, and of its fragments obtained after digestion with the enzymes (RFLP), was compared with a marker of 50 bp (DNA ladder GeneRuler, #SM0371, ThermoFisher, USA) or 100 bp (DNA ladder GeneRuler, #SM0241, ThermoFisher, USA). Image analysis was performed with a UV transilluminator (Slimline Series; Spectroline), the image was captured with an image digitizer (Enduro™ GDS, Labnet International, Inc., USA). Both the confirmation of the size of the amplified by PCR and the analysis of the RFLP were carried out with the TotalLab 1D software, version 14.0.

### Molecular techniques for the detection of pathogens

The PCR, nested-PCR or RFLP techniques were carried out according to the suggestion of the references for each of the parasites to be studied. For each molecular test the optimal concentration of primers, deoxyribonucleotide triphosphates (Dntps; Thermo Scientific, Cat. R0191, USA), Magnesium chloride (MgCl^2^; Thermo Scientific, Lot. 00603943, USA) and DNA polymerase (DreamTaq, DNA polymerase; Thermo Scientific, Cat. EP0702, USA) were adjusted and standardized to achieve a single amplification of the size suggested by the literature. For each of the molecular techniques performed, the sensitivity of the test was determined based on the minimun quantity required of DNA (sensitibity A) and the fewest number of life forms that the test detected (sensitibity B). Sensitivity A was performed from factor 10 dilutions of a DNA sample of known concentration and extracted from a feces sample whose positivity was verified by parasitological examination. Sensitivity B was performed from serial dilutions, by factor 10, of a fecal sample with a known count of vegetative forms, cysts or oocysts, followed by DNA extraction. The digestions with the restriction enzymes were performed at a final volume of 25 μL, adjusted with nuclease-free water, BE 1X buffer (Buffer CutSmart, BioLabs, New England, USA). Simple or double digestion was performed with 10 units of each of the fast-digesting restriction enzymes (BioLabs, New England, USA) and 5 to 10 μL of the amplification obtained by PCR at 37 °C/15 min. Table 1 summarizes the type of molecular technique used, the gene to amplify, the species to be defined, the name of the primers and their sequence, the size of the amplified, the type of restriction enzymes used, and the base references of each one of the standardized tests. Table 2 summarizes the final concentration for the primers, MgCl_2_, Dntps, Taq polymerase and the amplification program used for the molecular detection of *Giardia duodenalis*, *Entamoeba histolytica*/*E. dispar*, *Cryptosporidium* spp., *Cyclospora* spp. or *Eimeria* spp. and for *Blastocystis* spp. 

**Table 1.** Summary of the employed molecular techniques, the gene to be amplified, the species of parasites to be detected, the names of the primers and their sequence, the size of the amplified ones, the restriction enzymes and the bibliographic references of each test.

Parasite	Technique used	Amplified gene/Species	Primer name	Sequence of primers	Amplified size (bp*)	Restriction endonucleases	Reference
*Giardia duodenalis*	Semi-nested PCR- RFLP	Glutamate dehydrogenase (*gdh*)	GDHeF	5´-TCAACGTCAACCGCGGCTTCCGT-3´			21
			GDHiR	5´-GTTGTCCTTGCACATCTCC-3´			
			GDHiF	5´-CAGTACAACTCAGCTCTCGG-3´	432	*Bsp* I (*Nla* IV), *Rsa* I	
			GDHiR	5´-GTTGTCCTTGCACATCTCC-3´			
*Entamoeba histolytica/E. dispar*	Nested PCR	16S-like rRNA gene (genus)	EG-1F	5´-TAAGATGCACGAGAGCGAAA-3´	887-898		24
			EG-2R	5´-GTACAAAGGGCAGGGACGTA-3´			
		*E. histolytica*	EH-1	5´-AAGCATTGTTTCTAGATCTGAG-3´	439		
			EH-2	5´-AAGAGGTCTAACCGAAATTAG-3´			
		*E. dispar*	ED-1F	5´-TCTAATTTCGATTAGAACTCT-3´	174		
			ED-2R	5´-TCCCTACCTATTAGACATAGC-3´			
*Cryptosporidium* spp.	Nested PCR-RFLP	18S SSU rRNA (genus)	CrypF1	5′-TTCTAGAGCTAATACATGCG-3´	1,325		25
			Crypr1	5′-CCCTAATCCTTCGAAACAGGA-3´			
			CrypR2	5′-GGAAGGGTTGTATTTATTAGATAAAG-3´	826 - 864	*Ssp* I y *Vsp* I	
			CrypF2	5′-AAGGAGTAAGGAACAACCTCCA-3´			
*Cyclospora* spp. or *Eimeria* spp.	Nested PCR-RFLP	18S SSU rRNA	F1E	5′-TACCCAATGAAAACAGTTT-3´	636		26
			R2B	5′-CAGGAGAAGCCAAGGTAGG-3′			
			F3E	5′-CCTTCCGCGCTTCGCTGCGT-3′	294	*MnI* I	
			R4B	5′-CGTCTTCAAACCCCCTACTG-3′			
*Blastocystis* spp.	PCR- RFLP	18S SSU rRNA	SR1F	5´-GCTTATCTGGTTGATCCTGCCAGTAGT-3´	1780	*Himf* I, *Rsa* I	27
			SR1R	5´-TGATCCTTCCGCAGGTTCACCTA-3´			
*Blastocystis* spp.	Nested PCR	18S SSU rRNA	b11400	5´-GGAATCCTCTTAGAGGGACACTATACAT-3´	310		29
			b11710	5´-TTACTAAAATCCAAAGTGTTCATCGGAC-3´			

*Base pairs

**Table 2.** Summary of the final concentration of the primers, MgCl_2_, Dntps, Taq polymerase and the amplification program used for the molecular detection of *Giardia duodenalis*, *Entamoeba histolytica*/*E. dispar*, *Cryptosporidium* spp., *Cyclospora* spp., *Eimeria* spp., *Blastocystis* spp. and the bibliographic references for each test.

				Final concentrations per reaction					Cycling program temperatures				
Parasite	Primer name	Primers (µM)	MgCl_2_ (mM)	Dnps (µM)	Taq polymerase (Units)		Initial denaturation	Denaturation	Aneeling	Extensión	Cycles	Final extension	Ref.
*Giardia duodenalis*	GDHeF	0.5	3.0	200	2.0		94°C/4 min	94°C/45 s	55°C/30 s	72°C/45 s	35	72°C/7 min	21
	GDHiR												
	GDHiF	0.5	3.0	200	1.5								
	GDHiR												
*Entamoeba histolytica*/*E. dispar*	EG-1F	0.3	1.5	280	1.5		94°C/2 min	94°C/60 s	56°C/60 s	72°C/90 s	30	72°C/7 min	24
	EG-2R												
	EH-1	0.3	1.5	200	1.5		94°C/2 min	94°C/60 s	48°C/60 s	72°C/90 s	30	72°C/7 min	
	EH-2												
	ED-1F	0.3	1.5	200	1.5								
	ED-2R												
*Cryptosporidium* spp.	CrypF1	0.5	3.0	200	2.0		94°C/3 min	94°C/45 s	55°C/45 s	72°C/1 min	35	72°C/7 min	25
	Crypr1												
	CrypR2	0.5	3.0	200	1.25								
	CrypF2												
*Cyclospora* spp. or *Eimeria* spp.	F1E	0.5	1.0	200	1.5		95°C/5 min	92°C/30 s	53°C/30 s	72°C/90 s	35	72°C/10 min	26
	R2B												
	F3E	0.5	1.0	200	1.25				60°C/30 s				
	R4B												
*Blastocystis* spp.	SR1F	0.25	3.0	200	1.25		94°C/3 min	94°C/40 s	57°C/60 s	72°C/2 min	35	72°C/10 min	27
	SR1R												
*Blastocystis* spp.	b11400	0.5	3.0	200	2.0			94°C/60 s	60°C/60 s	72°C/1 min			28
	b11710												

## Results

**Figure 1.**
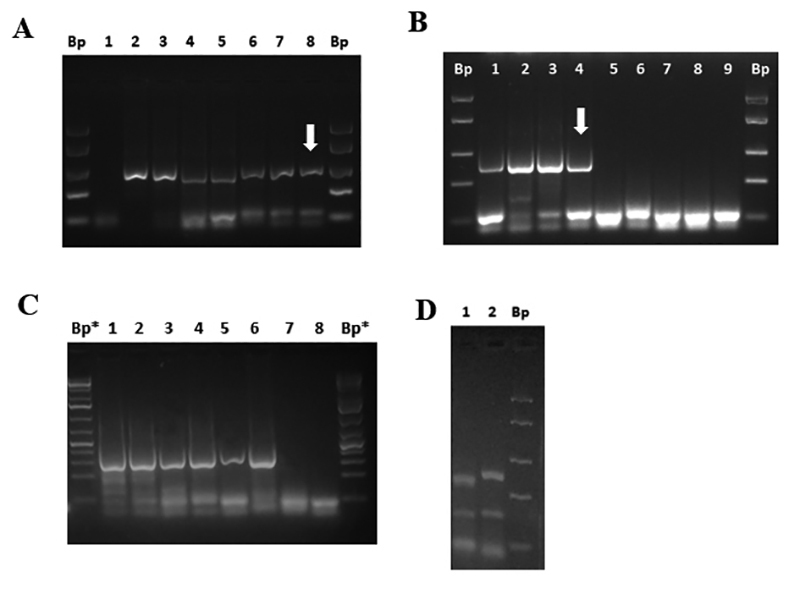
Detection and molecular characterization of the gene encoding *G. duodenalis* glutamate dehydrogenase (*gdh*) by means of nested semi-PCR and RFLP. (A) DNA detection limit (sensibility A). Lanes: (1) mix control; (2) 1 ng/µL DNA; (3) 0.1 ng/µL DNA; (4) 0.01 ng/µL DNA; (5) 1 pg/µL DNA; (6) 0.1 pg/µL DNA; (7) 0.01 pg/µL DNA; (8) 1 fg/µL DNA. (B) Cysts (Q) detection limit (sensibility B). Lanes: (1) 1 x 10^5^ Q; (2) 1 x 10^4^ Q; (3) 1 x 10^3^ Q; (4) 1 x 10^2^ Q; (5) 1 x 10^1^ Q; (6) 1 x 10^0^ Q; (7) 1 x 10^-1^ Q; (8) 1 x 10^-2^ Q; (9) mix control. (C) Molecular detection of the *G. duodenalis gdh* gene in human or animal samples. Lanes: (1) #33h (1 ng/µL; 2.4 x 10^5^ Q/mL); (2) #33h (0.1 ng/µL); (3) #19d (3.7 ng/µL; 7500 Q/mL); (4) #22d (0.19 ng/µL; 12500 Q/mL); (5) #25d (1.98 ng/µL; 1481 Q/mL); (6) #26d (1.72 ng/µL, uncounted); (7) #24d (0.1 ng/µL; 2222 Q/mL); (8) mix control. (D) Molecular characterization by RFLP. Lanes: (1) digestion with *Nla* IV; (2) digestion with *Rsa* I. Bp: 50 bp molecular marker (#SM1103; GeneRuler™, ThermoScientific, USA); Bp*: 75 bp molecular marker (#SM1113; GeneRuler™, ThermoScientific, USA). h: human; d: dog. Arrows indicate the limit of detection.

To amplify the gene encoding *Giardia duodenalis* glutamate dehydrogenase (*gdh*) of approximately 432 bp, a semi-nested PCR was performed. Under the same conditions of amplification, a DNA segment of 343 ± 24 bp was amplified, which coincided with the 318 bp reported in the literature [21], (Figure 1). It was possible to amplify DNA 1 fg/µL which was equivalent to 10 fg/rx (Figure 1A; Table 3) and 100 Q/mL (Figure 1B; Table 3). Figure 1C shows the amplification of the *G. duodenalis gdh* gene from different animal and human stool samples donated to Basic Research Laboratory. The DNA concentration range in these samples was found to be between 0.1 to 4 ng/µL of DNA and between 1,500 to 240,000 Q/mL. Figure 1D shows the RFLP obtained from the feces of a dog. RFLP is compatible with a BIII genotype, since fragment polymorphisms were obtained after digestion with *Nla* IV of 283 bp, 138 bp and 57 bp and with *Rsa* I of 310 bp, 137 bp and 36 bp [20-23]. 

**Figure 2.**
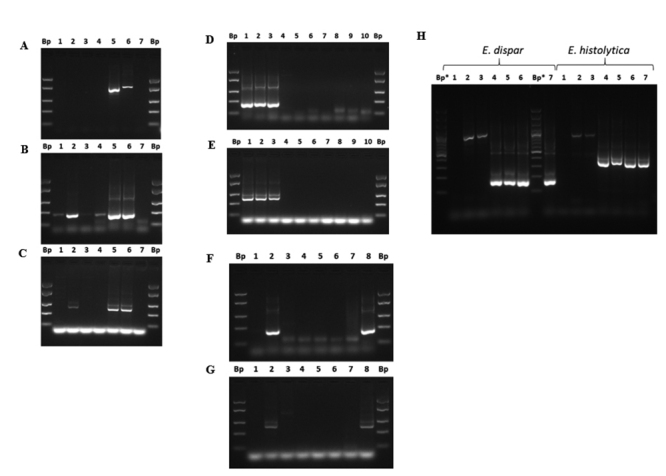
Molecular detection of the genus *Entamoeba* spp. and species *E. dispar* and *E. histolytica* by nested PCR. (A) PCR of the genus *Entamoeba* spp. (B) Nested PCR for *E. dispar*. (C) Nested PCR for *E. histolytica*. Lanes: (1) HL-67 (219 ng/µL); (2) HL-67 (21.9 ng/µL); (3) HL-68 (64.7 ng/µL); (4) HL-68 (6.47 ng/µL); (5) HL-27 (0.5 ng/µL); (6) HL-27 (0.05 ng/µL); (7) mix control. (D) Sensibility A for nested PCR to *E. dispar*. (E) Sensibility A for nested PCR to *E. histolytica*. Lanes: (1) 0.5 ng/µL DNA; (2) 0.05 ng/µL DNA; (3) 5 pg/µL DNA; (4) 0.5 pg/µL DNA; (5) 0.05 pg/µL DNA; (6) 5 fg/µL DNA; (7) 0.5 fg/µL DNA; (8) 0.05 fg/µL; (9) 1 atg/µL DNA; (10) mix control. (F) Cysts detection limit (sensibility B) for nested PCR *E. dispar*. (G) Cysts detection limit (sensibility B) for nested PCR *E. dispar*. Lanes: (1) 5000 Q; (2) 500 Q; (3) 50 Q; (4) 5 Q; (5) 0.5 Q; (6) 0.05 Q; (7) mix control; (8) positive control. (H) Nested PCR to *E. dispar* and *E. histolytica*. Lanes: (1) mixing control; (2) *E. coli* cysts (HL #35; 1.7 ng/µL); (3) *E. coli* cysts (HL #35; 0.17 ng/µL); (4-7) cysts of *E. histolytica* and *E. dispar* - lane (4) HL #27 (0.5 ng/µLDNA); (5) 0.05 ng/µL; (6) HL #27 (1.86 ng/µL); (7) HL #27 (0.186 ng/µL). Bp: 50 bp molecular marker (#SM1103; GeneRuler™, ThermoScientific, USA). Bp*: 100 Pb marker (#SM0323; Thermofisher, USA). HL: washed human feces. Arrows indicate the limit of detection.

The molecular detection of the genus *Entamoeba* spp. and the species of *E. histolytica* and *E. dispar* was carried out through a nested PCR, which amplified the gene that encoded the 16S-like rRNA, as described by Khairnar and Parija [24]. The external PCR that detected the genus of *Entamoeba* spp. performed with primers EG-1F and EG-2R, gave an amplification of approximately 887-898 bp (Figure 2A). Two nested PCRs were performed, one that detected *E. histolytica* with an amplification of 439 bp, with the primers EH-1F and EH-2R (Table 1), and another that detected *E. dispar* with an amplified 174 bp with the primers ED-1F and and ED-2R (Table 1). Nested PCR for *E. dispar* and *E. histolytica* resulted in amplification of 180 ± 6.5 bp (Figure 2B) and 386 ± 16.9 bp (Figure 2c), respectively, according to the literature [24]. According to these described conditions, it was possible to amplify 5 pg/µL of DNA or 12.5 pg DNA/rx PCR (sensitivity A), both for the nested PCR for *E. dispar* (Figure 2D) and for *E. histolytica* (Figure 2E) and up to 500 cysts/mL (sensitivity B) (Figure 2F; Table 3). It was possible to detect the presence of other species of the genus *Entamoeba* spp., amplified with the genus PCR, such as *Entamoeba coli* (Figure 2H). 

**Figure 3.**
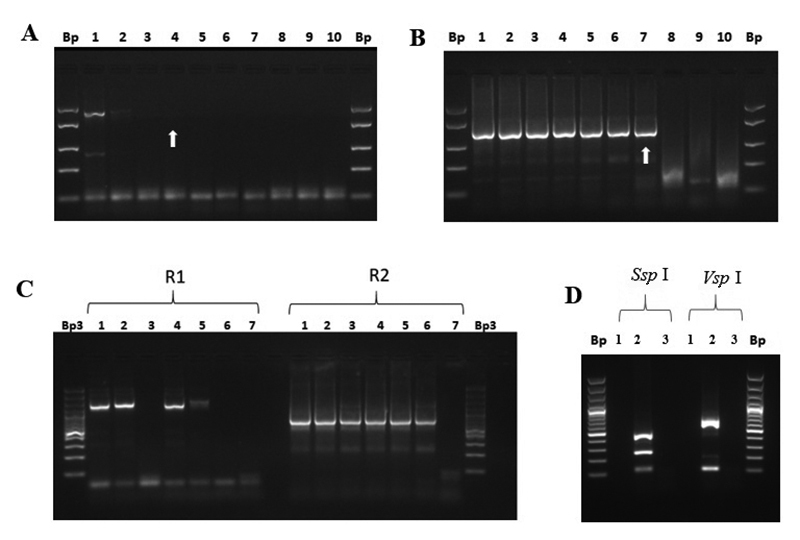
Detection and molecular characterization of the genus of *Cryptosporidium* spp. (A) External PCR: amplification of a 1460 ± 135.2 bp DNA segment. (B) Internal PCR: amplification of a DNA segment of 762 ± 38.3 bp. Lanes: (1) 10 ng/µL; (2) 1 ng/µL; (3) 0.1 ng/µL; (4) 0.01 ng/µL; (5) 1 pg/µL; (6) 0.1 pg/µL; (7) 0.01 pg/µL; (8) 1 fg/µL; (9) 0.1 fg/µL; (10) mix control. (C) External PCR (R1) and internal PCR (R2), sample human feces. Lanes (1-3): pure DNA (1.64 ng/µL), diluted 1/10 and 1/100, respectively. Lanes 4-6: pure DNA (3.61 ng/µL), diluted 1/10 and 1/100, respectively. Lane (7) mix control. (D) Molecular characterization of genotype B bovine *C. parvum* by RFLP with restriction enzymes *Ssp* I and *Vsp* I. Lanes (1) digestion reaction; (2) mix control. Bp: 50 bp molecular marker (#SM1103; GeneRuler™, ThermoScientific, USA); Bp3: 100 bp molecular marker (#SM0323; ThermoScientific, USA). Arrows indicate the limit of detection.

**Figure 4.**
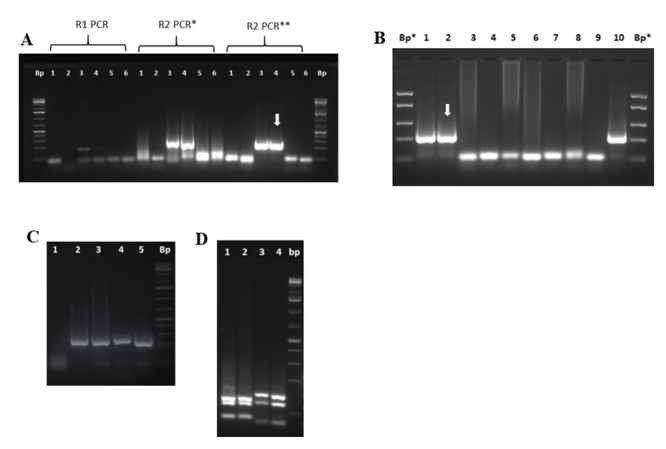
Detection and molecular characterization of the genus *Cyclospora* spp. or *Emeria* spp. (A) Standardization of nested PCR using 2.5 µL (*) and 5 µL (**) of external PCR with a human HL sample with *Cyclospora cayetanensis*. Lanes: (1) mix control; (2) 4.5 ng/µL; (3) 0.45 ng/µL; (4) 0.045 ng/µL; (5) 4.5 pg/µL; (6) 0.45 pg/µL. (B) Qocysts (Q) detection limit (sensibility B). Lanes: (1) 7,5 x 10^3^ Ooq.; (2) 1 x 10^3^ Ooq.; (3) 1 x 10^2^ Ooq.; (4) 1 x 10^1^ Ooq.; (5) 1 x 10^0^ Ooq.; (6) 1 x 10^-1^ Ooq.; (7) 1 x 10^-2^ Ooq.; (8) 1 x 10^-3^ Ooq.; (9) mix control; (10) positive control (human feces with *Cyclospora* oocysts). (C) Nested PCR (R2). Lanes: (1) mix control; (2) and (3) pure DNA (0.276 ng/µL) and diluted 1/10 of a sample of chicken feces with *Eimeria* spp., respectively; (4) and (5) pure DNA (1.64 ng/µL) and diluted 1/10 of a human stool sample with *C. cayetanensis*, respectively. (D) RFLP made with the enzyme *MnI* I. Lanes (1) and (2) *Eimeria* spp. RFLP; (3) and (4) *Cyclospora* spp. RFLP. Bp: 75 bp marker (#SM1331; Thermofisher, USA). Bp*: 50 bp molecular marker (#SM1103; GeneRuler™, ThermoScientific, USA). Arrows indicate the limit of detection.

To detect *Cryptosporidium* spp. and molecular characterization of *Cryptosporidium parvum* genotypes, a nested PCR was developed for the molecular detection of the genus by detecting the SSU 18S rRNA and RFLP gene for the identification of different genotypes of *C. parvum*, as published by Xiao *et al*. [25]. The amplification conditions (Table 2) were identical to those recommended in the literature [25], and allowed to amplify a DNA segment for the genus of 1359 ± 73 bp (Figure 3A) and 762 ± 38.3 bp for *C. parvum* (Figure 3B). Serial 10-fold dilutions of a DNA sample resulted in a nested PCR sensitivity for genus of 10 fg/µL that was equivalent to 50 fg DNA/rx (Figures 3A and 3B; Table 3). Figure 4C shows the molecular detection of *Cryptosporidium* spp. in a human stool sample demonstrating an increase in the level of detection in internal PCR at lower dilutions of DNA. Restriction enzyme digestion from nested PCR amplicon resulted in a polymorphism of 444 bp, 272 bp, and 130 bp after digestion with *Ssp* I, and 629 bp and 125 bp after digestion with *Vsp* I (Figure 3D), which coincided with *C. parvum* Bovine genotype B according to the literature [25].

The differential molecular detection between the genus of *Cyclospora* spp. and *Eimeria* spp. was carried out through a nested PCR by detecting the SSU 18S rRNA gene and RFLP with the restriction enzyme *MnI* I, according to what was published by Orlandi *et al*. [26]. The external amplification (R1) was performed with the pair of primers F1E and R2B and the internal one with F3E and R4B (Table 1). According to the amplification conditions for nested PCR (Table 2), a 284 ± 7.1 bp DNA fragment was amplified (Figure 4A), according to the literature [26], with a sensitivity A of 0.045 ng/µL or 225 pg/rx PCR (Figure 4A, Table 3), sensitivity B of 1000 oocysts (Figure 4B, Table 3). The nested PCR indiscriminately amplifies the genus of *Cyclospora* spp. and *Eimeria* spp. (Figure 4C) which may be different after performing the RFLP. The amplifications obtained were digested with *MnI* I, which allowed for the differentiation of the genus of *Cyclospora* spp. from the genus *Eimeria* spp. Three segments were obtained for the genus of *Cyclospora* spp. (133 bp, 104 bp, and 42 bp) and for *Eimeria* spp. (123 bp, 106 bp, and 61 bp) (Figure 4D), which coincided with the literature [26].

**Figure 5.**
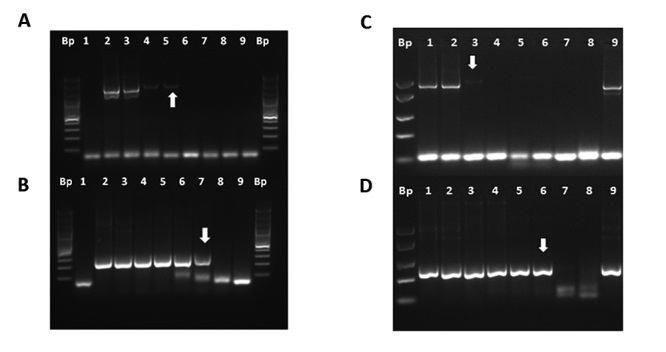
Molecular detection of *Blastocystis* spp. (A) DNA detection limit for PCR 1780 bp. (B) DNA detection limit for nested PCR 310 bp. Lanes: (1) mix control; (2) 0.16 ng/µL; (3) 0.016 ng/µL; (4) 1.6 pg/µL; (5) 0.16 pg/µL; (6) 0.016 pg/µL; (7) 1.6 fg/µL; (8) 0.16 fg/µL; (9) 0.016 fg/µL. Bp: 100 bp molecular marker (#SM0323; Thermofisher, USA). (C) Detection of vegetative forms (vf) of *Blastocystis* for PCR of 1780 bp. (D) Detection of vf of *Blastocystis* for nested PCR 310 bp. Lanes: (1) 3.6 x 10^5^ vf; (2) 3.6 x 10^4^ vf; (3) 3.6 x 10^3^ vf; (4) 3.6 x 10^2^ vf; (5) 3.6 x 10^1^ vf; (6) 3.6 x 10^0^ vf; (7) 3.6 x 10^-1^ vf; (8) mix control; (9) positive control (CL-MMC). Bp: 50 bp molecular marker (#SM1103; GeneRuler™, ThermoScientific, USA). Arrows indicate the limit of detection.

The detection and molecular characterization for *Blastocystis* spp., was carried out following the amplification conditions by detecting the SSU 18S rRNA gene, described by Yoshikawa *et al*. [27], were summarized in Table 1 and 2. With this protocol, a 1780 bp DNA fragment was amplified detecting the SSU 18S rRNA, which was consistent with the literature [27]. This protocol made it possible to amplify DNA from washed stool pellets (HL) or washed pellets (CL) from stool cultures in MBDM. The PCR for *Blastocystis* spp. had a detection limit of 160 fg/µL of DNA (sensitivity A), equivalent to 800 fg of DNA/RX PCR (Figure 5A; Table 3), and a sensitivity B of 3600 vegetative forms (Figure 5C; Table 3). To increase the sensitivity of PCR, nested PCR was performed that amplified a 310 bp DNA segment that detects the SSU 18S rRNA, described by Stensvold *et al.* [28]. This nested PCR increased the sensitivity by 2 orders of magnitude to 1.6 fg/µL of DNA (sensitivity A), which was equivalent to 8 fg DNA/rx PCR, (Figure 5B; Table 3) and the detection of approximately 4 vegetative forms of the parasite, which corresponded to 3 orders of magnitude lower (sensitivity B) (Figure 5D, Table 3). 

**Figure 6.**
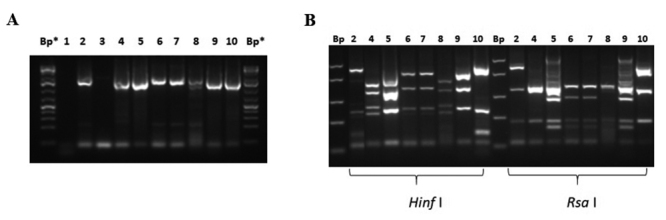
Molecular characterization of *Blastocystis* spp. by PCR-RFLP. (A) PCR amplification of 1780 Bp from human (h) and pig (p) from HL (washed feces) or Boeck culture washed pellet (CL). (B) RFLP and subtypes (Sts) obtained with the enzymes *Hinf* I and *Rsa* I. Lanes: (1) control mix; (2) HL-11 (h; St3); (3) HL-12 (h); (4) CL-15 (p; St4); (5) CL-13 (h; St1); (6) CL-31 (h; St1); (7) CL-32 (h; St1); (8) CL-36 (p; St4); (9) CL-12 (h; St1); (10) CL-11 (h; St3). Bp*: 75 bp molecular marker (#SM1113¸ Thermofisher, USA); Bp: 50 bp molecular marker (#SM1103; GeneRuler™, ThermoScientific, USA).

The molecular characterization for *Blastocystis* spp. was carried out by RFLP the enzymes *Rsa* I and *Hinf* I, according to the suggestions of Yoshikawa *et al.* [27]. These tests made it possible to discriminate the main genotypes that infect man (genotypes 1, 2, 3 and 4). The RFLPs obtained with each digestion were compared with those published by Yoshikawa *et al.* [27]. Figure 6A shows the PCR amplification of 1780 bp from DNA extracted from human or pig stool samples (HL or CL) and in Figure 6B shows the RFLPs for subtypes (St) 1, 3 and 4 can be observed, whose polymorphism coincides with that published by Yoshikawa *et al.* [27]. Of the 7 samples processed, some of them in duplicate (HL and CL), a polymorphism for St1 and St3 was obtained from human stool samples and a polymorphism for St4 for samples from pigs.

The Table 3 summarizes the sensitivity of the standardized molecular techniques, in terms of the minimum concentration used to obtain a good performance amplified (sensitivity A), and in terms of the minimum number of vegetative forms, cysts or oocysts of protozoa detected by the molecular techniques used (sensitivity B).

**Table 3.** Summary of the sensitivity of the different molecular techniques used to standardize the molecular detection of the parasites.

Parasite	Technique used	Expected amplified size (bp)	Sensitivity		
			Sensitivity A (ADN/µL)	Sensitivity A (ADN/rx)	Sensitivity B (protozoa/rx)
*Giardia duodenalis*	Semi-nested PCR-RFLP	432	1 fg/µL	10 fg	100^β^
*Entamoeba histolytica* and *E. dispar*	Nested PCR	887-898	5 pg/µL	12.5 pg	500^β^
*Cryptosporidium* spp.	Nested PCR-RFLP	826-864	10 fg/µL	50 fg	ud
*Cyclospora* spp. or *Eimeria* spp.	Nested PCR-RFLP	294	45 pg/µL	225 pg	1000^γ^
*Blastocystis* spp.	PCR- RFLP	1780	160 fg/µL	800 fg	3600^α^
*Blastocystis* spp.	Nested PCR*	310	1.6 fg/µL	8 fg	4^α^

*PCR nested from the 1780 bp of *Blastocystis* spp; α: vegetative forms; β: cysts; γ: oocysts; ud: undetermined.

## Discussion

Parasitological investigations of stool samples, in drinking water or food consumed raw or undercooked food, are widely used strategies in the research of parasitic infections in humans and animals or for investigation of infection routes [29]. The investigation of the different stages of protozoa or chromist through molecular biology techniques based on the investigation of the detection and characterization of parasitic DNA [30], from different matrices (water, feces, meat, soil), would improve the sensitivity and increase the probability of detection due to its high sensitivity and specificity [9]. In addition, it solves speciation problems, especially for those parasites species that are morphologically indistinguishable, as is the example for *Cryptosporidium* spp. [31] or *Blastocystis* spp. [32], whose species or genotypes would be impossible to differentiate through the parasitic diagnosis. Besides, some subtypes, assemblages or genotypes of *Blastocystis*, *Giardia duodenalis*, *Cryptosporidium* spp., could be present in animals [33]. Therefore, the detection and molecular characterization of these protozoa, which can be found in animals or in environments and waters contaminated with animal feces, so that the molecular investigation of these protozoa could collaborate with the molecular epidemiology of protozoa with zoonotic potential [34].

In this work, molecular techniques were standardized and tested to determine human pathogens, protozoa, and chromist. For all standardized molecular techniques (PCR, nested or semi-nested PCR, PCR-RFLP), the sensitivity of the technique was given in terms of the minimum quantity of DNA per µL or per PCR reaction (ADN/rx). The minimal quantity of biological forms detected by molecular tests, was called sensitivity B. Standardized tests allowed to find the species of the same genus, indistinguishable by microscopy, as is the case of the differentiation between *E. histolytica* and *E. dispar,* or arrive at genotypes (*Blastocystis* spp.) or assemblages (*G. duodenalis),* important for applying transition studies or molecular epidemiology.

The molecular detection of *G. duodenalis* in vegetable and fruit sediments has been reported through different methodologies, by direct microscopy via lugol [35], by fluorescence [36] or by molecular biology techniques [37]. However, only molecular biology techniques can differientiate the genotype to which *G. duodenalis* belongs, which allows for determining the source (animal or human) of its origin. In this work, a semi-nested PCR for the molecular detection of *G. duodenalis* was used, reporting a sensitivity of one (1) fg/µL of DNA, which was 2000 times lower than that reported by Read *et al*. [21]. When evaluating the detection limit of the forms of resistance, a detection limit of 100 cyst/rx was obtained for *G. duodenalis*, which was 100 times lower than that described by Read *et al*. [21], and detected one trophozoite of *G. duodenalis*/rx. This is probably due to the use of pure cultures of *G. duodenalis* trophozoite cultures [21]. Moreover, the use of stool samples and the presence of interferents can influence over PCR sensibility´s [38], although the samples used in this study were previously washed. In addition, the extraction of DNA from the cyst has a greater degree of difficulty, due to the presence of constituent chitin of the wall of the cyst [39]. Therefore, genotypes or assemblages of *G. duodenalis* research, using biomolecular technologies, would allow to make an inference of the parasite, which varies between hosts [40, 41] and between geographic areas [37]. In this work, the detections of a BIII genotype was possible, using the RFLP from DNA amplified by a semi-nested PCR after digestion with endonuclease *Nla* IV and *Rsa* I, whose polymorphism coincided with other reports [21-23].

The cysts of *Entamoeba* spp. can be detected with light microscopy depending on their morphological characteristics [42, 43], with the help of Lugol [44, 45]. *Entamoeba histolytica* (pathogenic amoeba) cysts are morphologically indistinguishable from *E. dispar* (commensal amoeba) and *E. moshkovskii,* considered free-living until it was isolated from a resident of Laredo, Texas, [46], who presented weight loss, epigastric pain and diarrhea. Other molecular epidemiological studies, through that the based on the determination of ribosomal SSU similar to 16S and the use of a multiplex PCR according to Khairnar and Parija [24], have revealed the presence of *E. moshkovskii* as responsible for gastrointestinal symptoms [47-49]. Therefore, determining the species is a critical step for the establishment of a treatment, when the pathogen is detected in human feces samples, or for indicating the origin of the contamination, if it is detected in vegetables or fruits for human consumption. In this study, nested PCR for the detection of *E. histolytica* and *E. dispar* was able to detect 500 cysts in washed stool samples (LH), well above that reported by Khairnar and Parija [24]. In comparison with our study, Khairnar and Parija [24] performed the experiments with trophozoite cultures, enriched and free from the remains of the stool samples. The nested multiplex PCR detection limit for *E. histolytica*, *E. dispar* and *E. moshkovskii* was found to be approximately 25 cells of *Entamoeba* protozoa, since 2.5 μL of template DNA (1000 parasites/100 μL of TE buffer), so it was expected that in our work the sensitivity B of the tests would be lower. On the other hand, the use of stool samples with cysts as a DNA extraction matrix can inhibit PCR by having a high amount of bacteria and detritus typical of digestion [38] and the cyst, with respect to the trophozoite, has a DNA extraction difficulty inherent in its conformation, such as a resistance structure [39].

*Cryptosporidium* spp. are protozoan parasites that infect humans and animals, and the second most common cause of diarrhea in children after rotavirus [50]. *Cryptosporidium* spp. it is characterized by its extensive genetic variation that results in the existence of 38 species and more than 60 genotypes of this parasite [51]. At least 20 different species cause moderate or severe infections in humans, of which *C. hominis* and *C. parvum* are the main causative agents [52]. Molecular tools have been developed to detect and differentiate *Cryptosporidium* spp. at the species/genotype and subtype level. These tools have been used increasingly to characterize the transmission of *Cryptosporidium* spp. in humans and animals [53]. In addition, they have also been used to investigate the sources of infection for humans, such as in water collections [54, 55] and in vegetables and fruits for human consumption. Genotyping tools based on DNA sequences of antigens and housekeeping genes identified genotype 1 for the human genotype and genotype 2 for the bovine genotype, within the *C. parvum* umbrela, gave rise to *C. hominis* and *C. parvum*, respectively, both infectious for immunocompetent and immunosuppressed people [56, 57]. 

In this work, it was possible to determine the sensitivity of nested PCR for the detection of 18S SSU rRNA for *Cryptosporidium* spp., at 10 fg/µL (equivalent to 50 fg DNA/rx). There is no literature reporting sensitivity A for nested PCR to detect this protozoan, but there are reports where this PCR allows amplifying 1 µL of DNA, without specifying the concentration of the DNA used [58]. In this study *Cryptosporidium* spp. oocysts were not quantified in this study, but it can be done in a Neubauer chamber after concentration by flotation in sucrose [59] or molecularly, when performing a quantitative or real-time PCR (q-PCR), where the number of copies per oocyst can be estimated according to the gene under investigation. In this regard, Li *et al.* [60] standardized the detection of oocysts of different *Cryptosporiridium* species and concluded that the amount of these parasites is determined by the fact that the gene used as a target has 20 copies per oocyst. Therefore, if real-time PCR can detect at least 20 copies of the gene, the sensitivity of the molecular test would be one oocyst. Real-time PCR would be a more sensitive technique and could quantify the copy number for a parasite like *Cryptosporidium* spp., which would be more convenient [61-63] than its detection after a flotation concentration in sucrose [59]. 

Regarding the simultaneous molecular detection for the *Cyclospora* spp. and *Eimeria* spp., it is possible to discriminate *Cyclospora cayetanensis* by RFLP only if the sample tested is from human feces. The detection of the genus of *Cyclospora* spp., in another type of sample, implies the presence of other species of animal origin (non-human primates) that could be present in the analyzed samples of sediment of vegetables, fruits or drinking water [64, 65], and although these are not pathogenic for humans, it would illustrate the contamination of cultures with non-human feces. Therefore, the molecular detection recommended for *Cyclospora* spp. would be sequencing of conserved genes such as those of SSU rRNA, after amplification by PCR [60], or using more specific primers that prevent the amplification of DNA from oocysts of another genus [66]. On the other hand, finding *Eimeria* spp. in sediment samples of fruits or vegetables for human consumption could mean the use of chicken manure [67], bovine [68] or equine manure [69], between others, as compost to fertilize vegetable or fruit crops, or the use of water contaminated with animal feces. Therefore, it would be important to identify the species to be able to identify the origin of this protozoan.

In this study, the limit of sensitivity for the detection of DNA for the genus of *Cyclospora* spp. or *Eimeria* spp. was 225 pg/rx. The minimal amount of oocysts of *Cyclospora* spp. or *Eimeria* spp. 1000 oocysts/rx were found to be 10 times less than the 100 oocysts of *Cyclospora* spp. or *Eimeria* spp., reported by PCR by Orlandi *et al.* [26]. In this regard, the samples used by Orlandi *et al.* [26] were kept in 2% potassium dichromate, which favored oocyst maturation. This could favor the sensitivity of the applied molecular technique, since a mature oocyst will have a greater amount of DNA than inmature ones, facilitating its finding through molecular methodologies. In this context, and during the standardization of molecular techniques for the detection of *Cryptosporidium* spp. in calves, Toledo *et al.* [59] demonstrated, that the amount of DNA required to detect the 18S rRNA gene [25], can be 16 times lower, when using an enriched sample of oocysts stored in potassium dichromate (0.025 ng of DNA) than from faeces (0.4 ng of DNA). Therefore, the sensitivity of a PCR will depend on how enriched and pure it is, which supports the decrease in sensitivity in some of the tests carried out in this work compared to the literature.

Regarding the molecular detection limit for *Blastocystis* spp., a detection limit of 3600 evolutionary forms per reaction was obtained by amplifying the SSUrRNA 18S gene of 1780 bp by endpoint PCR, as described by Yoshikawa *et al*. [27], but almost 4 evolutionary forms per reaction, when performing the nested PCR, whose endpoint PCR was described by Stensvold *et al.* [28]. When compared with references in the literature, Yoshikawa *et al*. [27] do not mention the parasite molecular detection limit. Stensvold *et al.* [28] reported a PCR sensitivity of 80 parasites per gram of feces, which was 20 times higher than that reported in this work, after using the PCR described by them as a nested PCR, from the amplified yield obtained by amplifying the 1780 bp SSUrRNA gene described by Yoshikawa *et al.* [27]. By molecularly characterizing *Blastocystis* spp. a predominance of St3 over St1 and St4 was obtained, all of them found as infectious subtypes (Sts) in man [70-72], and among the nine Sts present in man (St1 al St9) with St2 being the most common Sts found in human feces [72]. The RFLP performed allows defining the subtypes found in 95% of humans infected with *Blastocystis* [71]. Some of the Sts found have been related to gastrointestinal symptoms [71-73] and others to their absence [74] or as part of the intestinal microbiota [75].

## Conclusions

The sensitivity A (minimum quantity of DNA) of the standardized molecular techniques by PCR reaction was 10 fg, 12.5 pg, 50 fg, 225 pg 800 fg and 8 fg for *G. duodenalis* (semi-nested PCR), *Entamoeba* spp. (genus), *Cryptosporidium* spp., for the simultaneous genus of *Cyclospora* spp. and *Eimeria* spp. and for *Blastocystis* spp. after performing 1780 bp PCR or, nested PCR (310 bp), respectively. For the parasites in which it was possible, the minimum number of protozoa or chromists that were detected by the molecular technique used was determined, which was 100 and 500 cysts for *G. duodenalis* and *Entamoeba* genus, of 1000 oocysts for the detection of forms of *Cyclospora* spp. or *Eimeria* spp. and 3600 or four vegetative ones for *Blastocystis* spp. (PCR 1780 bp) or *Blastocystis* spp. (nested PCR; 310 bp), respectively. The molecular detection of protozoa and chromist was achieved and the molecular characterization allowed the genotyping of some of the parasites such as *Giardia duodenalis*, *Cryptosporidium* spp., and *Blastocystis* spp. For the molecular detection of *Cyclospora* species, it would be advisable to sequence conserved genes such as those of the SSU rRNA, after their PCR amplification [52] or to use more specific primers for avoid amplification of oocysts’ DNA of another genus [58]. For the detection of *Cryptosporidium* spp. gene sequencing is recommended. This study opens the door to the molecular epidemiology of intestinal protozoosis and *Blastocystis* spp., which can be used for epidemiological studies in humans, animals, sources of transmission and as diagnostic tools in countries where intestinal parasites are a public health problem. 
